# ESEM-EDX Mineralization and Morphological Analysis of Human Retrieved Maxillary Sinus Bone Graft Biopsies before Loading

**DOI:** 10.3390/jfb14070391

**Published:** 2023-07-24

**Authors:** Hideki Imai, Carlo Prati, Fausto Zamparini, Giovanna Iezzi, Daniele Botticelli, Maria Giovanna Gandolfi, Shunsuke Baba

**Affiliations:** 1 Department of Oral Implantology, Osaka Dental University, 8-1 Kuzuhahanazonocho, Hirakata 573-1121, Osaka, Japan; imaihideki@gmail.com (H.I.); baba-s@cc.osaka-dent.ac.jp (S.B.); 2 School of Dentistry, Department of Biomedical and Neuromotor Sciences, University of Bologna, 40125 Bologna, Italy; carlo.prati@unibo.it (C.P.); fausto.zamparini2@unibo.it (F.Z.); mgiovanna.gandolfi@unibo.it (M.G.G.); 3 Laboratory of Biomaterials and Oral Pathology, School of Dentistry, 40125 Bologna, Italy; 4 Department of Medical Oral and Biotechnological Sciences, University of Chieti, 66100 Chieti, Italy; gio.iezzi@unich.it; 5 ARDEC Academy, 47923 Rimini, Italy

**Keywords:** dental implants, ESEM-EDX, electron microscopy, retrieved implants, peri-implant bone, implant loading, mineralization

## Abstract

This study aimed to analyze the morphology of bone graft granules, the presence of granule demineralization, and bone morphology in retrieved human maxillary sinus bone graft biopsies. Healthy patients underwent sinus bone augmentation using lateral access. Two different dimensions of the antrostomy were performed, a 4 mm or 8 mm height. After 6 months, all sites received one implant using a flap technique, crestal positioning, and submerged healing. Implant biopsies were retrieved after 3 months and were histologically processed. The ESEM analysis was performed on the entire portion of the peri-implant bone (up to 750 µm from the implant thread). Three different regions of interest (ROIs) were selected: the coronal, middle, and apical portions of the implant. In these areas, EDX was performed, and calcium (Ca), phosphate (P), nitrogen (N), and their atomic ratios (Ca/P, Ca/N, and P/N) were calculated. Different bone tissue electron-dense areas were detected through grayscale intensity quantification of ESEM images with different organic (N) or inorganic (Ca,P) compositions. A total of 16 biopsies from 16 healthy patients were analyzed. Bone graft granules were mostly detected in the apical ROI. New bone tissue bridges were detected in the apical and middle ROI. These structures, with lower Ca/N and P/N ratios, were connected and enveloped the bone graft granules. Cortical ROI revealed the most mineralized bone tissue. Conclusions: After 9 months, bone graft resorption was only partially completed and new bone tissue appeared less mineralized in the middle and apical ROI than in the coronal ROI.

## 1. Introduction

Sinus floor elevation is often applied if the edentulous posterior segments of the maxilla do not have sufficient bone volume for implant installation [1,2]. Different biomaterials may be used for bone augmentation procedures. Autologous bone is still considered the gold standard, even though it presents some disadvantages such as limited availability and involvement of second surgical sites [3,4,5].

The histological healing processes of various biomaterials for maxillary sinus floor elevation has been studied [3]. A series of bioptic clinical studies have reported data on the integration of mini-implants installed six months after sinus floor elevation and retrieved three months later [6,7,8,9]. These studies showed a low impact of both dimension and position of the access window on osseointegration. Similarly, the use of a collagen membrane on the access window or subjacent the sinus mucosa did not influence final osseointegration. A large portion of the implant surface (2.5–30%) was in contact with the residues of the biomaterial, which negatively affected the osseointegration process. Additionally, it was also shown that implants with a turned surface presented significantly lower osseointegration compared to moderately rough surfaces (11.0% vs. 28.9%, respectively) [8]. These histological studies did not effectively evaluate mineralization processes around engrafted tissues and the surface modification of bone graft in the atrophic maxilla. Microanalysis of the peri-implant bone may give us an idea of the mineralization events that occur in such critical areas.

Environmental scanning electron microscopy (ESEM) used on histological samples is a non-destructive technique that allows sample microanalysis without any manipulation (dehydration of the metal coating as required by SEM). Energy dispersive X-ray spectroscopy (EDX) has been successfully used to analyze the microchemistry of bone tissue close to several bioactive materials in a previous animal model [10] and to analyze the inorganic and organic components of biological samples during new bone formation processes. This protocol allows a qualitative and semi-quantitative investigation of the constitutive elements of histological samples, providing further detailed information to those obtained with histological assessment. Bone tissue mineralization can be analyzed through the calculation of calcium, phosphate (from hydroxyapatite) and N = nitrogen (from collagen) levels and their atomic ratios. 

The aim of this study was to analyze the morphology of bone graft granules, presence of granule demineralization, and morphology of bone in retrieved human maxillary sinus bone graft biopsies.

## 2. Materials and Methods

The clinical procedures of the study were approved by the Ethical Committee of the University Corporation Rafael Núñez, Cartagena de Indias, Colombia (protocol #01-2015; 19 May 2015) and were performed at the same university. The Helsinki Declaration was respected, and informed consent was obtained from patients upon inclusion in the study.

### 2.1. Study Population

Twenty-four healthy volunteers, who desired fixed oral rehabilitation supported by implants in an edentulous distal region of the maxilla with insufficient volume of bone and consequently were in need of sinus floor elevation, were recruited in the present randomized controlled trial.

The evaluations in the present study were performed on biopsies from another study on sinus floor elevation and delayed implant installation. Cone-beam computed tomography (CBCT) and histological results have been reported elsewhere [6,11]. In these studies, sample calculation and inclusion and exclusion criteria were also reported. To be admitted to the study, the patients had to satisfy the following requisites:

(i) ≥21 years of age;
(ii) Edentulous zone in the posterior segment of the maxilla needed to be restored with an implant-supported fixed prosthesis;
(iii) Height of the sinus floor ~4 mm or less;
(iv) Good general health;
(v) No contraindications for oral surgical procedures;
(vi) Not pregnant.

The following exclusion criteria were adopted:

(i) Presence of a systemic disorder;
(ii) Chemotherapeutic or radiotherapeutic treatment in progress;
(iii) Smokers of >10 cigarettes/day;
(iv) Acute or a chronic sinusitis;
(v) Treated for bone augmentation in the region of interest.

Two groups were formed, including access windows that were either 4 or 8 mm in height. Patients were randomly assigned to one of the two groups (*n* = 12). 

### 2.2. Surgical Procedures

The surgical procedures have been reported in detail in previously published articles [6,11]. Briefly, after exposing the lateral bone wall of the sinus, access windows were prepared, either 4 mm or 8 mm in height. A sonic-air instrument (Sonosurgery TKD, Calenzano, Italy) was used. The subantral space was grafted with corticocancellous porcine bone (OsteoBiol Gen-Os, 0.250–1.0 mm, Trycare, Bradford, UK), and a collagen membrane (OsteoBiol Evolution, 0.3 mm, Trycare, Bradford, UK) was used to cover the sub antrostomy. After 6 months of healing, 2.4 × 8 mm mini-implants (Sweden & Martinam, Padova, Italy) were placed crestally. After three months, biopsies containing mini-implants were obtained.

### 2.3. Histological Processing

The biopsy specimens were fixed in 10% formalin, dehydrated in alcohol, incorporated in glycol-methacrylate resin (Technovit 7200 VLC, Kulzer, Hanau, Germany), and polymerized. Ground sections ~30 µm in width were prepared following the long axis of the mini-implants and were stained with acid fuchsin and toluidine blue.

### 2.4. ESEM-EDX Analyses

The ESEM-EDX microanalyses were performed following a validated protocol [12,13].

Histological samples were placed on the ESEM stub and examined without any previous preparation (uncoated specimens). The operative parameters were as follows: low vacuum 100 Pascal, accelerating voltage of 20–25 kV, working distance of 8.5 mm, and 133 eV resolution in quadrant backscattering detector mode (0.5 wt. % detection level, amplification time 100 μs, measuring time 60 s) [10,12,13]. Three different acquisitions in backscattered mode of the bone tissue were made at 100× magnification, corresponding to the coronal portion of the histological sample, the middle portion, and the apical portion.

Then, ESEM images at higher magnifications (from 500× to 3000×) were obtained in correspondence with the biomaterial granules. 

EDX analyses were performed in areas of approximately 30 µm ×30 µm in each ESEM image. The organic/inorganic content of the bone tissue and biomaterial granules was measured using EDX with ZAF correction, and the qualitative and semiquantitative element contents (weight % and atomic %) were evaluated. For all spectra, the presence of calcium (Ca), phosphorous (P), nitrogen (N), and their atomic ratios (Ca/P, Ca/N, and P/N) were calculated. Additional analyses of “as-received” corticocancellous porcine bone were performed to evaluate the microchemical and the morphological aspects before implantation.

ImageJ software (NIH software Version 1.54, Bethesda, MD, USA) was used to detect different bone areas with different electron densities. The grayscale intensity was then correlated with the EDX data following a previous methodology [14] applied to other histological biopsy analyses [12,13]. Different grayscale values indicate different mineralization values; the blackest areas indicate low mineralization, and the light gray areas indicate high mineralization. 

Differences in Ca, P, N, Ca/N, P/N, and Ca/P ratios were statistically analyzed using one-way ANOVA. The *p*-value was set at 0.05.

## 3. Results

Biopsies were obtained from 20 patients, 10 per group. Of these, four were excluded due to the detachment of the sample from the histological slides. As a result, 16 samples were analyzed through ESEM-EDX.

### 3.1. ESEM-EDX Analysis of Bone Areas and Bone Graft Granules

EDX analysis revealed several mineralization patterns in bone areas (Table 1).

Bone graft granules revealed intermediate mineralization values between those of bone area 3 (lamellar bone) and bone area 4 (cortical bone). New bone tissue/woven bone revealed lower mineralization, and, as a result, lower Ca/N and P/N values. Bone marrow areas (bone area 1) showed the lowest mineralization and were easily detected in histological samples. N showed similar values in all bone areas, with the exception of bone area 2, which had slightly higher values.

**Figure 1:**
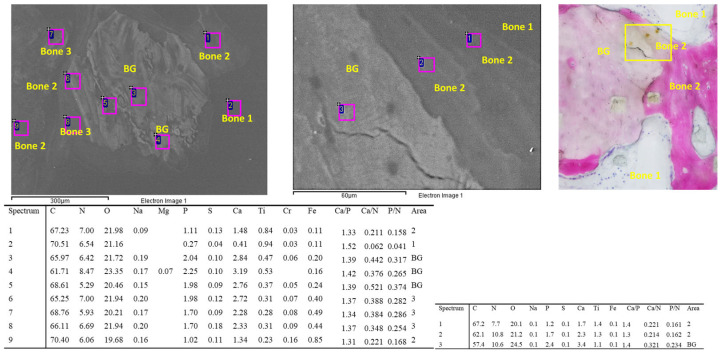
EDX analysis was performed on an ESEM image at 500× of one bone graft granule surrounded by bone tissue. All values are reported as atomic percentages. The analysis at 3000× magnification revealed a mineralization gradient from the bone graft (site 3, bone area 3) to the new bone-forming structures (site 2 and 1, bone area 2). Please note that the new bone tissue enveloped the mineral granule.

Representative images of bone granules are shown in Figure 1. The ESEM-EDX analysis at 500× revealed different bone mineralization trends around the bone graft granule, which appeared more electron-dense. The bone tissue was less mineralized and heterogeneous, resembling woven bone (bone area 2).

**Figure 2:**
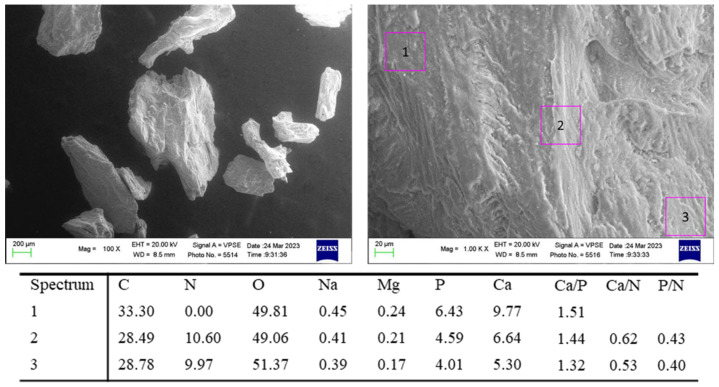
ESEM- EDX analyses of “as-received” bone graft granules used in the present investigation. EDX revealed a high Ca/P ratio, which was very similar to those of bone area 3 and 4. All values are reported as atomic percentages. Interestingly, Ca/N and P/N ratios were markedly higher when compared to all the bone areas, which was attributable to the markedly higher Ca and P content in the “as-received” biomaterial.

ESEM-EDX analysis of “as-received” granules are reported in Figure 2. The biomaterial was composed of particles ranging from 200 to 1000 µm with an irregularly shaped morphology. EDX was performed on the ESEM images at 1000× magnification. The analysis revealed the constitutional elements of the biomaterials. Generally, the Ca/P ratio was similar to bone 3 and 4 values (Ca/P ratio ranged from 1.32 to 1.51). Interestingly, markedly higher Ca/N and P/N ratios were reported. This increase was attributed to the significantly higher Ca and P content in the as-received biomaterials. N appeared quite constant, although in some cases (such as in spectrum 1) it was not reported, likely attributable to the absence of the organic portion (i.e., collagen) in this area.

### 3.2. Analysis of the Bone Tissues at Coronal, Middle and Apical ROI

Sixteen histological specimens were analyzed using OM and ESEM-EDX. The presence of bone graft granules showed marked variations when considering cortical versus apical and middle ROI. The bone tissue in contact with the granules was still immature. In most cases, bone area 2 was detected along with some bone area 3. Consequently, the bone tissue in the middle and apical bone was characterized as woven bone with low amounts of lamellar bone.

In cortical ROI, bone tissue was mostly compact with no or very low presence of biomaterial granules. High mineralization, detected by high Ca/N and P/N ratios, was observed (Bone Area 3 and 4). Low presence of bone area 2 was reported. In the middle and apical ROI, a high presence of bone area 2 was observed. 

**Figure 3:**
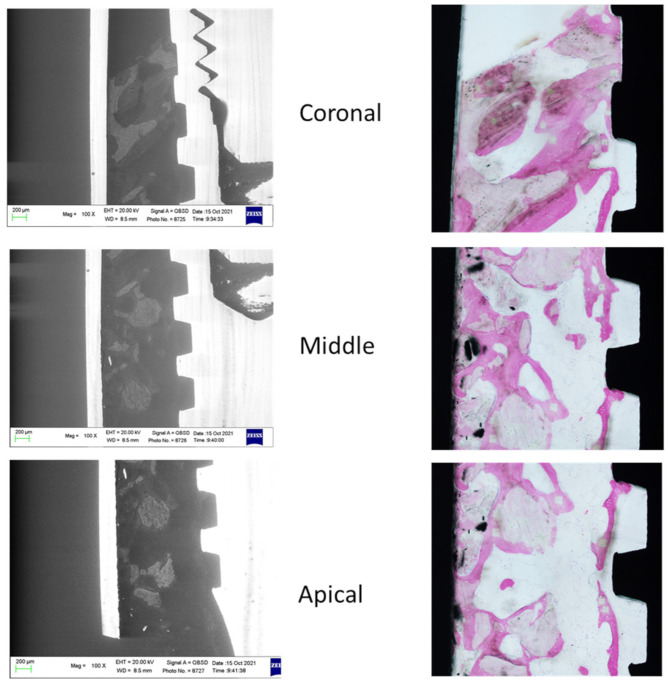
ESEM and OM images were obtained to identify bone tissue and bone grafts around the implant. Original magnification 100×.

**Figure 4:**
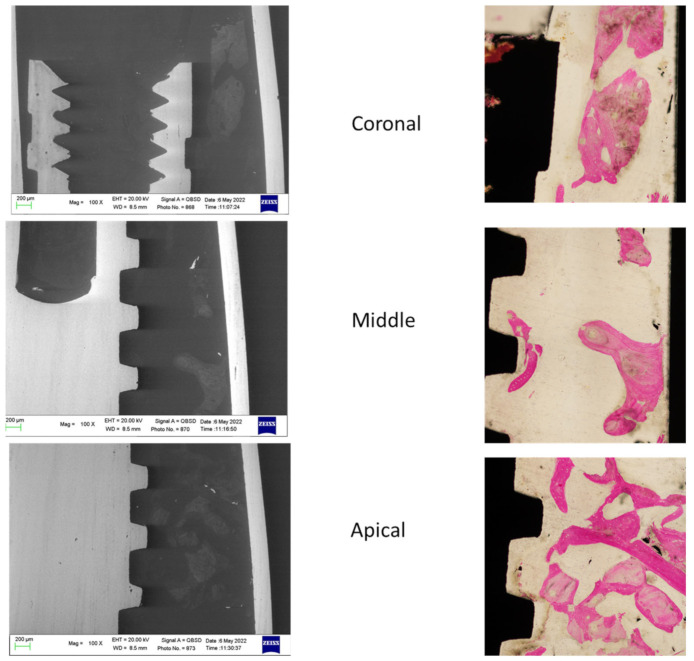
ESEM and OM images were obtained to identify bone tissue and bone grafts around the implant. Original magnification 100×.

The ESEM-EDX analysis at the coronal, middle and apical ROI of two specimens included in the study is schematized in Figure 3 and Figure 4.

### 3.3. Coronal ROI

ESEM analyses of the cortical ROI revealed highly electron-dense tissue close to the implant threads. The tissue appeared mature, with limited remodeling areas or osteon structures. Bone marrow areas were also identified in proximity to the threads. The bone tissue appeared as mature trabecular bone. 

**Figure 5:**
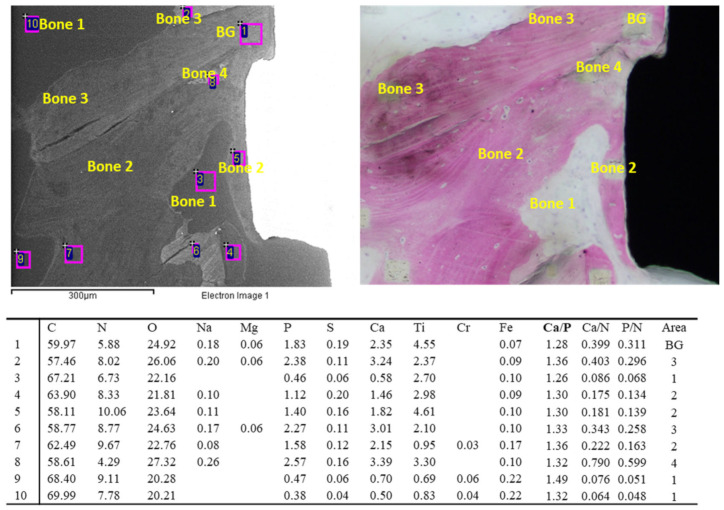
EDX analysis of the ESEM image at 500× on bone tissue located in the Coronal ROI. All values are reported as atomic percentages. In this area, bone tissue was mostly composed of bone area 3 and 2, with limited presence of low-mineralized bone (bone area 1). Traces of biomaterial granules (BG) were detected close to the implant thread (but were not in contact).

EDX analysis revealed that the tissue was mostly composed of bone area 3, with some traces of bone area 2 and bone area 1. Bone area 4 (the highest mineralized) was detected close to the implant thread (see Figure 5).

### 3.4. Middle ROI

**Figure 6:**
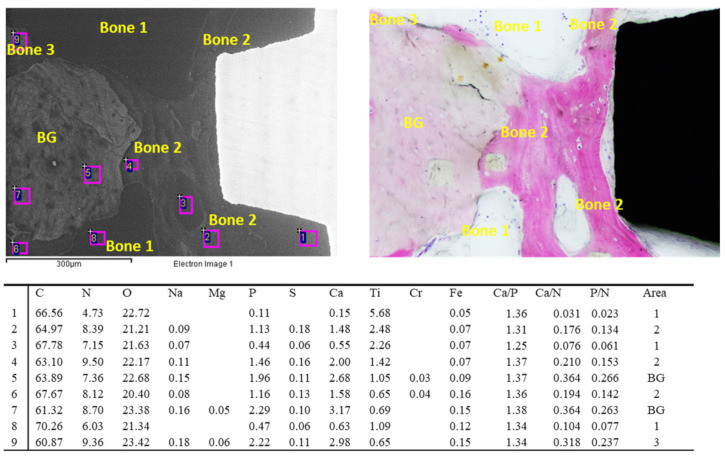
EDX analysis of the ESEM image at 500× on bone tissue located in the middle ROI. All values are reported as atomic percentages. Note that the bone graft granule is partially surrounded by new bone tissue and is connected to the implant thread through new bone tissue (sites 1–4, bone area 1 and 2).

ESEM images taken at one representative Middle ROI revealed a higher presence of low-electron-density tissues close to the implant thread (Figure 6). The image shows the presence of a large granule of biomaterial. EDX analysis revealed the presence of low-mineralized new bone tissue (bone area 2) that connects the implant thread and the biomaterial granule. The EDX analysis of the granule revealed higher mineralization.

### 3.5. Apical ROI 

**Figure 7:**
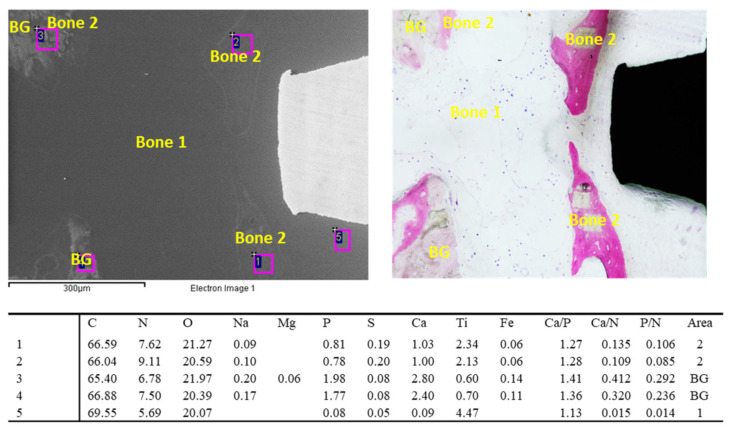
EDX analysis of the ESEM image at 500× on bone tissue located at the apical ROI. All values are reported as atomic percentages. The apical ROI was characterized of a high presence of Bone Area 1. The implant thread was not in contact to the new bone tissue and bone graft were observed at distant sites (more than 300µm).

ESEM images at the apical ROI revealed a very low-electron-density tissue with limited high-electron-density areas (Figure 7). EDX analyses revealed the presence of bone area 1 and some bone area 2 close to the implant thread. Biomaterial granules were detected far from the implant and were enveloped by new bone tissue (Bone Area 2).

### 3.6. Coronal ROI

**Figure 8:**
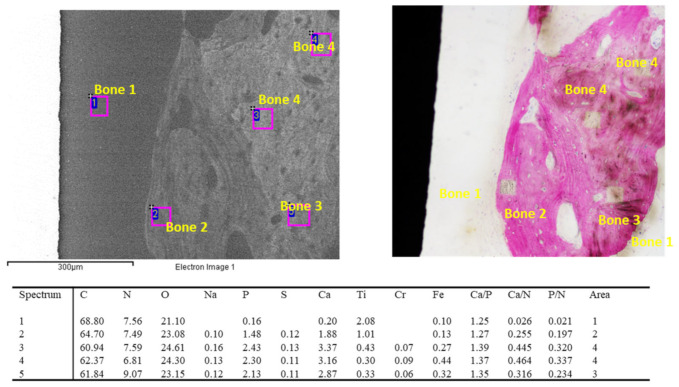
EDX analysis of the ESEM image at 500× on bone tissue located in the coronal ROI. EDX analyses showed high Ca/N and P/N atomic ratios at sites located >300 µm from the implant body. All values are reported as atomic percentages. The bone tissue appeared highly mineralized (sites 3–5, bone areas 3 and 4). Low Ca/N and P/N values were detected close to the implant (site 1, bone area 1), and at 200 µm (site 2, bone area 2). No traces of bone graft material were detected in this ROI.

The ESEM images in Figure 8 show the presence of compact, highly electron-dense tissue that was not in contact with the implant thread. EDX analysis revealed the presence of bone area 3 and 4 with high mineralization. Closer to the implant, low mineralized bone area 2 was also detected. Bone graft granules were not observed in this ROI.

### 3.7. Middle ROI

**Figure 9:**
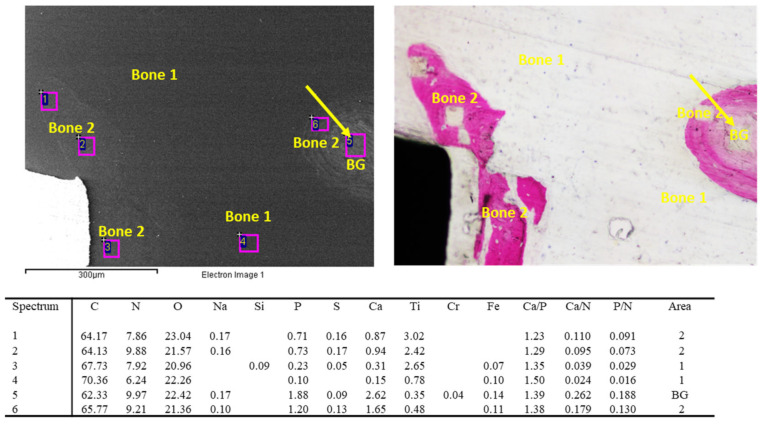
EDX analysis of the ESEM image at 500× on bone tissue located in the middle ROI. EDX analyses revealed low Ca/N and P/N ratios, representing bone tissue with low inorganic content (sites 1–4, bone area 1 and 2). All values are reported as atomic percentages. A high presence of bone marrow areas and some new bone tissue was identified close to the implant thread. The higher Ca/N and P/N ratios found at distant sites (sites 5 and 6, bone area 2 and 3) revealed the presence of a more mineralized structure (arrow), resembling partially resorbed bone graft particles encircled by new bone tissue.

The ESEM image of the middle ROI in Figure 9 shows a markedly low mineralized area with a high presence of bone area 1. Newly formed tissue (bone area 2) was detected on the top of the thread. One bone graft granule was observed at a site that was distant from the implant thread. The granule was surrounded by mineralized bone tissue. EDX analysis revealed low Ca and P (Ca/N and P/N) levels in the newly formed bone tissue. In contrast, the EDX analysis of the granules revealed higher Ca and P, and higher Ca/N and P/N atomic ratios.

### 3.8. Apical ROI

**Figure 10:**
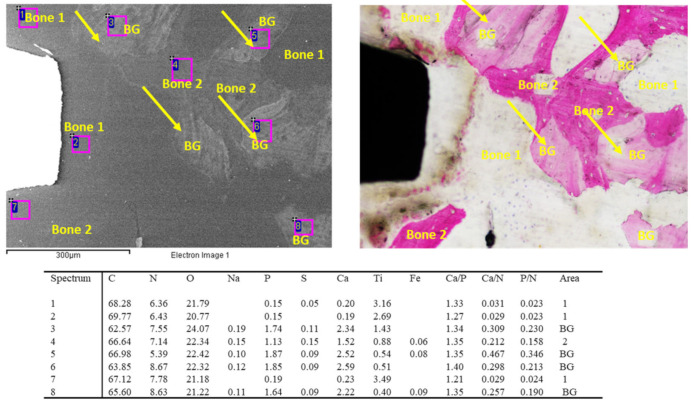
EDX analysis of the ESEM image at 500x on bone tissue located at the apical ROI. Numerous bone graft granules were identified in this ROI (arrows). EDX on the granules revealed high Ca/N and P/N values, which represent highly mineralized tissues (Sites 3, 5, 6). These granules are connected by less electron-dense tissue (site 4, bone area 2), which resembles a new bone. Low electron density and low mineralized tissue were found in contact with the implant body (sites 1, 2, 7, bone Area 1).

ESEM analysis of the apical ROI (Figure 10) revealed a large number of biomaterial granules surrounded by newly formed bone tissue. EDX analysis revealed bone area 2 and 3 close to the granules. In contrast, the bone tissue close to the implant thread was poorly mineralized and mostly composed of bone area 1 and 2.

## 4. Discussion

This study analyzed the mineralization and morphology of peri-implant bone around implants placed after bone augmentation procedures in the maxillary sinus. It was also possible to investigate the morphology of the bone graft granules and their micromorphological modifications after 9 months in real in vivo clinical conditions. 

The possibility of analyzing implants retrieved from the human maxilla after bone graft procedures and implant installation has rarely been reported in the literature [6,7,8,9,15,16,17,18,19]. The causes of implant retrieval in humans are usually attributable to implant fractures [15,16,17] or peri-implantitis [19]. In other cases, retrieval was performed post-mortem [18].

Two different approaches were used: a 4 mm antrostomy versus an 8 mm access windows. A previous histomorphometric study revealed no significant differences in the quantity of new bone formed in either group. In the previous investigation it was not possible to directly investigate the mineral composition of the bone grafts embedded in the bone tissue or the mineralization of the bone tissue around the granules or in contact with the implant [6]. Being a hierarchical material, the combination of material composition and the unique structural design (i.e., bone areas) determines the overall strength of bone and its quality, both critical conditions for long-term implant stability [15]. 

By calculating the atomic percentages of elements constituting inorganic bone (Ca and P from hydroxyapatite) and organic (N from collagen) tissues, it was possible to create a “map” of tissues with different electron densities and, consequently, different mineralization. The same technique has been used and validated in other histological studies, such as to assess bone mineralization around unloaded dental implants retrieved from the posterior mandible after 4 months [20] or around dental implants retrieved after more than 10 years [12]. Optical microscope images were used for comparisons to discriminate between the bone tissue, biomaterials, and bone marrow.

The ESEM-EDX analysis of the “as-received” biomaterial was used to detect significant differences in morphology and mineralization when implanted in sinus floor augmentation and maintained in situ for 9 months. The as-received material had a markedly higher percentage of Ca and P, and consequently higher Ca/N and P/N ratios. Morphological images revealed that the granules were smaller in size. EDX analysis of the implanted biomaterial revealed markedly lower mineralization. Mineralization ratios (Ca/N and P/N) of bone grafts detected in the histological samples were close to those of bone areas 3 and 4, demonstrating an incomplete resorption of these materials that could persist for a longer time at the surgical site. 

These data indicate a marked reduction in calcium and phosphate from the granule structure, which was probably leached and used as active signals for new bone formation at the surgical site. The biomaterial acts as a valid bio-interactive surface for bone-mineralizing cells such as osteoblasts. Progressive dissolution at the periphery of the bone graft by osteoclast cells leads to the additional release of bio-interactive signals that trigger new bone formation around these structures. In agreement with this, a previous study on porcine corticocancellous bone inserted into post-extractive alveoli revealed high biocompatibility and osteoconductivity with variable resorption rates [21]. 

Our investigation revealed a higher presence of new mineralized bone tissue that enveloped and wrapped the granules, creating a new bone bridge (Figure 10). This tissue was mostly composed of bone area 2 (with limited Ca and P but higher N) and limited bone area 3 (higher Ca and P values and similar N proportion compared to bone area 2). Most of the bone granules were detected in the middle and apical ROI and were the areas that were surgically augmented to place the dental implant in the atrophic sinuses. In contrast, the cortical ROI was generally composed of more mineralized tissue (bone area 4, which presented the highest Ca and P values). The high percentage of bone area 1 (bone marrow area), which is characterized by low Ca and P, was confirmed by the relatively low BIC% found in a previous histological study [6]. Moreover, the presence of a high rate of low-mineralized tissue suggests that complete healing requires a longer time.

The new mineralized bone tissue observed around the granules indicated a successful (yet not complete) integration of the biomaterial with the surrounding bone, supporting the formation of a stable but yet not functional implant site.

These findings highlight the clinical importance of proper biomaterial selection and surgical techniques to achieve successful osseointegration, ultimately leading to long-term implant success and improved patient outcomes. As a histological study on retrieved biopsies, the study limitation could be the lack of consecutive specimens to analyze the initial baseline (graft insertion) and the mineralization progress before implant insertion. With this in mind, the analysis of the “as-received” bone graft allowed us to compare and describe the mineralization differences at the moment of the histological processing (9 months). Future studies could include the analysis of bone graft modification at different end-points.

## 5. Conclusions

The present study revealed that after nine months, bone graft resorption was only partially completed. ESEM-EDX revealed that bone tissue was mostly composed of low-mineralized bone areas, in particular at the middle and apical ROI. A high percentage of new bone was observed in correspondence with the graft granules, which was highly interconnected by bone. New bone, however, appeared less mineralized at the middle and apical ROI when compared to coronal ROI. The presence of bone area 4, detected at the cortical region, indicated the establishment of a strong and mineralized interface between the bone and the dental implant only in this area. Globally, these findings show that bone tissue is not suitable for clinical load-bearing applications. Bone mineralization requires additional time to achieve a complete osteointegration.

## Figures and Tables

**Table 1:** EDX analysis of bone areas and bone grafts in the histological samples according to Ca, N, P and their atomic ratios (Mean ± SD). Different letters represent statistically significant differences (*p* < 0.05). Bone area 1, bone marrow areas; bone area 2, woven bone around granules; bone area 3, lamellar bone around granules; bone area 4, old cortical bone; BG, bone graft granules.

	Bone Area 1	Bone Area 2	Bone Area 3	Bone Area 4	BG
Ca/N	0.05 ± 0.13A	0.17 ± 0.08B	0.32 ± 0.13C	0.45 ± 0.13D	0.39 ± 0.07C
P/N	0.03 ± 0.09A	0.13 ± 0.13B	0.24 ± 0.15C	0.32 ± 0.19D	0.28 ± 0.05C
Ca/P	1.32 ± 0.08A	1.35 ± 0.08A	1.35 ± 0.08A	1.39 ± 0.08A	1.38 ± 0.02A
Ca	0.48 ± 1.07A	1.6 ± 1.02B	2.83 ± 0.8C	3.15 ± 1.09C	3.02 ±0.29C
P	0.36 ± 0.7A	1.2 ± 0.7B	2.08 ± 0.7C	2.25 ± 0.36C	2.19 ±0.22C
N	8.4 ± 1.7A	9.08 ± 1.78A	8.74 ± 1.7A	7.18 ± 1.8B	8.01± 2.01A

## Data Availability

Data are available upon reasonable request.
